# Historical Retrospect for January, 1825

**Published:** 1825-01

**Authors:** 


THE LONDON
### Medical and Physical Journal.

No 1 OF VOL. LIII.] JANUARY, 1825. [No 311.

For many fortunate discoveries in medicine, and for the detection of numerous errors, the world is indebted to the rapid circulation of Monthly Journals; and there never existed any work, to which the Faculty, in Europe and America, were under deeper obligations, than to the Medical and Physical Journal of London, now forming a long, but an invaluable, series.—RUSH.

### HISTORICAL RETROSPECT

### FOR JANUARY, 1825.

SOME of our readers are of opinion that our Retrospects are too long, and might, perhaps, be discontinued; while others give us to know that they think this the most useful part of the Journal. To abide by the former advice, would assuredly save us more labour than gentlemen unacquainted with the burthen of editorship are, perhaps, aware of; but we are inclined to think "in medio virtus," and therefore purpose to abridge this department, without altogether expunging it. We recollect the fable of the old man and his ass, who, attempting to please all, failed to please any, too well, to hope that, by any arrangement, we can give universal satisfaction: yet we trust, while we thus avoid details of any considerable length, that an outline of some of the most important works or papers, which our limits may have prevented us from previously noticing, together with a reference to the sources of fuller information, will prove acceptable. Teeming as the press now is with medical periodicals, both at home and abroad, it is impossible for any work, with restricted limits, to notice, in a satisfactory manner, every remarkable case or novel doctrine: we, therefore, purpose to give only those, *not already mentioned, which appear of some importance*, either as individually interesting, or as referring to the general state and progress of medical science. In doing this, most frequent reference will be made to the labours of our continental neighbours, both because they are more voluminous in themselves, and in general less accessible to English readers.

### ANATOMY.

The long-promised work of M. SERRES, on the Comparative Anatomy of the Brain in the four classes of Vertebrated Animals, has at length made its appearance.* In this work, a rapid sketch is given of the general laws of organization, principally in order to apply two favourite doctrines,—that of *symmetry*, as the principle of the development of double organs; and that of *conjunction*, as the principle of their re-union. After some consideration relative to the mode of development of the fundamental parts of the encephalon, in which we find nothing that has not been previously advanced by BLUMENBACH, MECKEL, and TIEDMANN, in Germany, and M. GEOFFROY ST. HILAIRE in France, the author endeavours to determine the primitive state of the brain in all the classes. He finds the cerebro-spinal axis *(l'axe cerebro-spinal,)* always constituted, in each of the classes, of four elementary parts,—namely, the spinal marrow; two rounded bulbs, corresponding to the tubercula quadrigemina; two bulbs anterior to these, which are the rudiments of the cerebral hemispheres; and two transverse plates, situated posteriorly, and which are the first vestiges of the cerebellum. If any cause should arrest the development of one or more of these parts, the encephalon may nevertheless be formed with the characters of the class which is inferior. So much for the identity of the composition of the brain in all the classes generally. The relative degrees of development of the different parts in each of the different classes, is thus stated:—

In *fishes*, the optic lobes are the predominating part; the cerebral hemispheres are very small; the olfactory lobe is very considerable; the cerebellum is moderately developed.

In *reptiles*, the optic lobes lose their influence; the cerebellum almost disappears; the hemispheres of the brain are much more developed than in fishes; whilst the optic thalamus is, in its turn, comparatively small.

In *birds*, the cerebellum is the predominant part; the optic lobes are diminished; the cerebral hemispheres are enlarged; while the optic thalami are almost lost.

In the *mammalia*, the cerebral hemispheres become, in their turn, the predominant organs; the cerebellum continues its transverse development; the tubercula quadrigemina are reduced to their smallest form.

The olfactory lobule experiences great variety, being very much developed where the hemispheres are small, while it diminishes, and almost disappears, in proportion as we advance from the ruminating to the carnivorous animals, and from the monkey tribe to man.

According to M. Serres, the proportion of white medullary matter increases with the increasing development of an organ;

* *Anatomie comparée du Cerveau dans les quatre Classes d'Animaux Vertebrès, appliquée à la Physiologie et à la Pathologie du System Nerveux.* Par E. R. SERRES, D.M.P.—Paris, 1824.

while the reverse holds good as it becomes diminished, the grey matter now in its turn predominating: hence he concludes, along with Dr. Gall, that the white matter is the principal seat of nervous agency.

The phenomena attending the development and complete formation of the encephalon, in the four classes of animals above alluded to, are minutely and elaborately considered. The principle on which depends the formation of the different parts of the nervous system, is to be found in the blood-vessels. First, says M. Serres, the arteries of the spinal marrow appear, then those of the brain, and lastly those of the cerebellum; this being the relative order in which the parts become developed. A remarkable phenomenon is presented in the opposite march of the cerebrum and cerebellum: it depends upon this, that the internal carotids, by the influence of which the hemispheres are developed, are directed from before backwards; while the vertebral arteries, which form the cerebellum, on the contrary, have their course from behind forwards. From an extensive examination of the phenomena in question, M. Serres concludes that a relation always exists between the development of the arterial branches and of the corresponding parts of the encephalon; so that, according to this author, we ought to be able, on being made acquainted with the sanguiferous system in any animal, to deduce therefrom the condition of the nervous system,—or, at least, of the encephalon.

We confess that we have received, from the production of M. Serres, no very favourable impression of his good faith; and it appears to us, from the manner he is spoken of in some of the foreign Journals, that he is not held in high estimation among his countrymen. Why has he passed over in silence the labours of Reil, the inquirer who, we hesitate not to say, paved the way for much of the investigation which has since been made, and who actually described many parts of the brain, and still more of the cerebellum,—the discovery of which MM. Gall, Spurzheim, and others, not only claim, but obtain the credit and reputation due to another.

Those interested in this kind of investigation, will find a paper worthy of perusal recently published by M. Rolando:* it relates principally to the development of the different parts. He neither regards the brain as a bulbous growth from the spinal marrow, nor the spinal marrow as a prolongation of the brain, as has been the fashion to do.

For extensive and interesting details respecting the Nervous System in various classes of the lower animals, we beg to refer

* *Annali Universali*, July 1824.

our readers to the researches of MM. DESMOULINS,* BAILLY,† and TREVIRANUS;‡ or to the account of those given by CUVIER, in a report laid before the Royal Academy of Science.§

Every thing deserves attention which tends to facilitate the acquisition of anatomical knowledge,—especially at present, when so many obstacles occur in procuring bodies for dissection. We have formerly spoken favourably of the plates now publishing by CLOQUET in France, and by Mr. LIZARS in this country; and we have to mention, in addition, a work more splendid than either, that of Dr. ANTOMMARCHI.|| These plates represent the parts of their natural size, under different aspects and in successive layers: to use the words of M. DUMERIL, in his report to the Academie des Sciences, they are "*de panoramas anatomiques.*" They are coloured or in black, and so contrived that any three corresponding plates may be joined, so as to represent the human figure of its full stature. The price of this splendid work is certainly moderate, being twenty-five francs for each fasciculus in black, and three times that sum for those which are coloured. The fasciculus contains six plates, three complete and three in outline, with letters of reference.

---

### PHYSIOLOGY.

In our preceding Retrospect, in reviewing the state of physiology, and the manner in which it is at present cultivated, we remarked, that "the French will not believe that we see with our eyes or hear with our ears, unless it be proved by experiment." This may possibly have appeared to some of our readers to have been an expression rather too extravagant, and we are free to confess that we did not anticipate its being so speedily fulfilled to the very letter. Nevertheless, we find it recorded in the last Number (July) of MAGENDIE'S Journal, that he has cut the optic nerve, with the remarkable effect of destroying vision. "Quoi qu'il en soit, le nerf (viz. the optic,) etant coupé d'un coté la vue m'a semblé immediatement abolie de ce coté;" and again, "si la section est faite sur les deux nerfs l'animal est evidemment aveugle,"—a result which our readers will not hear without astonishment!

---

* *Recherches Anatomiques sur le System Nerveux des Poissons;* par M. DESMOULINS.

† *Recherches sur le System Nerveux dans les quatre Classes d'Animaux Vertebres;* par M. BAILLY.

‡ *Journal Complementaire.*

§ *Analyse des Travaux de l'Academie Royale des Sciences, pendant l'année* 1823; par M. CUVIER.—Paris, 1824.

|| *Planches Anatomiques du Corps Humain, executées d'après des Dimensions naturelles, accompanées d'une Texte explicatif;* par le Dr. ANTOMMARCHI. Publiées par M. le Comte DE LASTEYRIE.—Paris, 1824.

The truth of the matter appears to be, that M. Magendie has become aware of the untenable positions laid down in his former paper, and is desirous of backing out of the difficulty with as good a grace as may be. In the paper now before us, he says, "With regard to smell, I have nothing to add to what I stated in my former Memoir upon this sense. The tri-facial being once divided, all trace of sensibility disappears; no odoriferous body, either at a distance or in contact,—not even corrosives, affect the pituitary membrane in any manner. *This does not prove, as I have said before, that the seat of smell is in the branches of the fifth pair;* but it proves, at least, that the olfactory nerve indispensably requires the branches of the fifth pair to enable it to act; that it is destitute of general sensibility, and that it can only have a special sensibility relative to the odoriferous bodies." We believe this to be a true statement of the influence of the fifth pair over the sense of smell; but the form of expression is entirely different from that made use of in the former Memoir, in which, in allusion to the same set of experiments, he says, "The result of this experiment, in counterproof of the preceding, appears to me to show that the sense of smell, as far as regards powerful odour, *depends upon the branches of the fifth pair*, and that the first pair of nerves has *no share with the fifth* in bestowing this function." Now, we ask, would it not be more ingenuous to acknowledge that he finds his first ground untenable, and so abandons it, than thus attempt to reconcile such direct contradictions.

The same remarks apply to vision. We observed, in our review of his opinions above alluded to, that "the only circumstance in this paper which renders the opinion that vision depends upon the fifth pair plausible, is the contraction of the iris, and its remaining insensible to light;" and argued, nevertheless, that this insensibility was no satisfactory proof of the absence of vision, or, at least, of the optic nerve receiving the peculiar stimulus of light, although the apparatus by which it usually manifests such an impression was paralysed; observing, that, "in some amaurotic eyes, the iris contracts on the application of light," this being just the converse of the former position. Accordingly, we find these views corroborated by M. Magendie's own statement; for it appears, after all, that, when the fifth pair has been divided, very strong light (as that of the direct rays of the sun) is perceived,—the animal shutting the eyelid to exclude it: and this certainly not only confirms our conjecture with regard to the eye, but goes far to convince us that the same thing would hold good with the sense of smell, but for the insuperable barrier formerly pointed out,—viz. that it "is not evinced by external manifestations."

In his preceding Memoir, as we have seen, M. Magendie, speaking of the sense of smell, asserts, "that the first pair of nerves has no share with the fifth in bestowing this function." Of hearing he says, "Observation has led me to believe that the division of the fifth pair likewise entails the loss of hearing:" and with regard to vision, although no expression equally strong is to be found, yet the general object of his experiments was undoubtedly to prove that this sense likewise depended on the fifth pair; so that, to use his own words, "the general theory of sensations would require to be reformed." Now, however, his language is, that "the fifth pair exerts a very great influence on smell, sight, and hearing: that it is the organ of taste and of general sensibility of the face, and the different cavities connected with it." This is exactly the view which we took of the subject, in our analysis of M. Magendie's former Memoir. We argued that the fifth pair gave common sensibility to the membrane of the nose, as to all the neighbouring parts, and consequently must have an influence on the organ to which it is sent; and with regard to the eye, we remarked, in allusion to the impaired vision produced by dividing the fifth pair, "that it is a very complicated piece of apparatus, which requires it to be in a perfect state in order to exercise its function." Magendie has not done us the honour to take any notice of our criticism of his doctrines; but we are glad to find, as we have shown above, that he now virtually, if not verbally, agrees with us in our former statement,—"that, however satisfactory his experiments may be so far as they go, yet they are not such as to warrant the conclusions he has drawn from them."*

While on this subject, we may mention that Mr. C. BELL has just published a detailed exposition of his doctrines, illustrated by various diagrams.† This work has the advantage of developing his views in a regular and systematic manner, and must satisfy all unprejudiced persons of the plagiarisms which we have so frequently pointed out. We have already, on repeated occasions, entered so fully into the opinions of Mr. C. Bell with regard to the nerves, that it would be a work of supererogation to do so now: such of our *readers* as have *read* the Journal, must be pretty well acquainted with them.

Others among the French, who follow the example of Magendie without his talent, continue their pursuit of science by the repetition of innumerable experiments upon living animals: and, to make assurance doubly sure, seem invariably to proceed upon the presumption that nothing, of any kind, hi-

---

* *Journal de Physiologie*, July; and Appendix to the Number of this Journal for July, 1824, p. 92.

† *An Exposition of the Natural System of the Nerves of the Human Body; with a republication of the Papers delivered to the Royal Society on the subject of the Nerves.* By CHARLES BELL, &c. &c.—London, 1824.

thereto ascertained, ought to be admitted till verified under their own eyes; forgetting that, if human testimony is thus to be rejected, their own opinions must fail to command assent beyond the limited arena of their cruel and bloody operations. We shall never cease to raise our voices against the revolting and unscientific method of investigation at present pursued in cultivating physiology in France; and, so far as the influence of this Journal extends, we shall do our utmost to prevent its adoption in this country. We do not make any affected or canting pretensions to fine feeling;—we do not object to experiments upon animals, where such experiments are calculated to elucidate important truths;—we appreciate as much as any the invaluable researches of the Hunters, and, in our own days, those of Mr. Brodie on the Poisons, methodically planned, and regularly conducted, each having an object, and each being required to give integrity to the whole. M. Magendie, in his last Number, defines science to be—facts, *(d'ailleurs les faits sont la science;)* and this, no doubt, is all the science of the mere experimentalists; but Haller and Hunter, whose claims to physiological reputation will scarcely be disputed, regarded facts only as they proved the sources of useful inductions,—the materials, of which the Temple of Science was to be constructed. We still fear that, to some of our readers, we may appear to carry this matter too far. One word more in illustration, and we have done.

In the last Number of Magendie's Journal, to which we have above referred,—the Journal of Physiology, *par excellence*,—we find it gravely stated, that tying the aorta immediately above its bifurcation into the common iliacs, causes weakness of the lower limbs, ("si sur un chien, on lie l'artère aorte immediatement au dessus de sa bifurcation en iliaques primitives, l'animal laisse bientôt remarquer une faiblesse des extremités posterieures," &c.;) that, if a part be lacerated, it does not bleed so freely as if it be cut with a sharp instrument,—a fact, no doubt, new to all practical surgeons, ("si pour l'ablation d'un rein, ou de la rate on coupe les vaisseaux de ces organs avec l'instrument tranchant, et qu'on en neglige la ligature il survient une hemorrhagie abondante, et suivie d'une mort prompte: tandis que la déchirure des mêmes vaisseaux par l'arrachment des mêmes viscères, ne donne lieu qu'a l'écoulement des très petite quantité de sang, et rend la ligature superflue.") We shall say nothing of the cruelty of tearing out the spleen and kidneys of these unfortunate animals, to prove what all the world knew, but pass on to another extraordinary discovery,—which is, that, if the windpipe of any animal of the class mammalia be very thoroughly stopped, the said animal perishes of asphyxia; or, to use a less learned form of expression, dies for want of breath. (Que l'on lie, sur un cone solide la trachée artere d'un mammifère, de manière à intercepter toute communication entre les bronches et l'air exterieur, l'animal perit d'asphyxie en un temps determiné, quoique variable selon l'age qu'il porte, le genre auquel il appartient, &c.") The inquiry, however, does not terminate here, but is continued in order to show that, if the thorax or abdomen be opened, death does not take place quite so rapidly ; the only practical application of which that we can see is, that, in case capital punishments accompanied with torture should ever again be introduced, the victims condemned to be hanged might linger somewhat longer, if the hint thrown out by M. Segalas d'Etchepare were practically adopted.*

M. Savart has published the results of numerous experiments, made with a view of ascertaining the uses of the membrana tympani and internal parts of the ear. Referring to the paper itself for the details,† we must content ourselves with enumerating the conclusions at which he has arrived. They are—1st. That the communication of vibrations by means of air appears to observe the same laws, at least with respect to minute distances, as those which govern solids. 2d. It is not necessary to suppose, as has been done heretofore, the existence of a particular mechanism to make the membrana tympani always vibrate in unison with the bodies which act upon it. 3d. That the degree of tension does not vary, except to augment or diminish the amplitude of its excursions *(excursions)*, as supposed by Bichat; but with this difference, that M. Savart's experiments prove the reverse of Bichat's conjecture, which was, that the membrane was relaxed by powerful impressions, and became tense from those which were feeble. 4th. That the vibrations of the membrane are communicated without alteration to the labyrinth, by the medium of the ossicula, as the vibrations of the upper table of a musical instrument are transmitted to the lower. 5th. That the ossicula likewise modify the extent of movement in the vibrating parts contained in the labyrinth. The author further thinks that the extent of the membrane of the tympanum in different animals influences the number of sounds which they can perceive, and likewise the limits at which these sounds commence, or cease to be perceptible to the animal.

A paper of some interest has been published on the Eye, by M. Mondini, of Bologna. It relates chiefly to the pigmentum

---

* *Note sur quelques Points de Physiologie;* par M. Segalas d'Etchepare.—Journal de Physiologie, 3 Numero, 1824.

† *Journal de Physiologie,* No. 2, 1824.

nigrum, which he asserts to be properly no pigment, but a true membrane. The black colour, he affirms, is owing to the presence of minute particles of oxid of iron, which may be separated by the loadstone.*

Dr. Knox, whose researches in comparative anatomy have procured for him a place among the most industrious naturalists of the day, has published some remarks on the lacteal system in the testaceous mammalia, which are of importance, both as they disprove a position of the celebrated Tiedman, and as they bring us back to an old doctrine, which had been invalidated by the assertions of the naturalist above mentioned. Tiedman had been led to suppose that, in some animals, as the seal, the chyle having been conveyed to the mesenteric glands by the common lacteals, (viz. the vasa afferentia,) was there absorbed by veins only, and thus conveyed directly into the blood, without passing along the thoracic duct; and, consequently, that this duct was destined, in all such animals, for the transmission of lymph alone. Dr. Knox has succeeded in demonstrating the vasa *efferentia* as well as *afferentia*, and showing, by a preparation deposited in the museum of the College of Surgeons in Edinburgh, that they may be injected with quicksilver. Thus, we return to the established opinion, that there exists, in all animals with which we are acquainted, a peculiar set of vessels, whose proper business it is to convey into the blood the nutrient fluid derived from the food.†

Dr. Francini has performed a series of experimental investigations relative to Absorption, of which the following are the most important results:—1. That the lymphatic vessels of the intestines are those which absorb the chyle. 2. That it is not demonstrated that they absorb any other fluid in the intestinal cavity. 3. That there is no solid argument tending to prove that the lymphatics of other parts are absorbents. 4. That it is certain that the veins of the intestines, of the abdomen, and bronchiæ, absorb. 5. That experiment has not made it obvious whether the lymphatics or veins absorb the fluids existing in the cavities of the cellular tissue, &c. 6. That, from numerous experiments, both in the state of health and disease, it is probable that the absorption of fluids in the cavities is, in a great measure, performed by sanguiferous vessels.‡

The Lymphatic System has likewise been the subject of study with M. Lauth, who has favoured the world with an account

* *Oper. Scient. dell Univer. de Bologna.*
† *Observations on the Anatomy of the Lacteal System in the Seal and Cetacea;* by Dr. Knox, &c.—Edinburgh Med. and Surg. Journal, July 1824.
‡ *Opuscol. Scient. di Bologna*, No. 1.

of his investigations; and it is to the general results alone that we can afford space to draw the attention of our readers. They differ very essentially from those above mentioned, and are—1st. That the lymphatics absorb. 2. That these vessels terminate partly in the veins, partly in the tissue of the different organs, and in the glands. 3. That there appears always to be certain substances in them, which are poured into the veins to be more speedily eliminated. 4. That nothing proves the absorption of veins; which, indeed, is contradicted by the idea we entertain of this class of vessels. M. Lauth likewise rejects the idea of any absorption by means of transudation by inorganic pores. It is proper to state, that this author defines a lymphatic to be any vessel arising from an open mouth, or extremity, terminating in a vein, and endowed with the power of absorbing.

The subject of Generation has recently received considerable attention, if not much elucidation, from several physiologists. The observations to which we allude are principally directed to the spermatic animalculæ. The existence of these is asserted, by M. St. Vincent, to admit of ready demonstration, by taking the testicle of an animal recently dead, and making a puncture in it; the semen oozes out, and a small portion of it is to be examined through a microscope. The animalcules are so numerous, that they scarcely have room to move; but, if the semen be diluted with water, they become separated, and are more easily subjected to scrutiny. Their resemblance to tadpoles is said to be striking. Various other particulars are detailed in the paper from which these remarks are taken.*

MM. Prevost and Dumas have likewise instituted experiments connected with this curious subject.† They deny the influence of the aura seminalis, deem the spermatic animalculæ necessary, and hold that the semen must be actually introduced into the uterus in order to produce impregnation. If this be so, there are some anomalies to be explained.

The subject of Muscular Contraction has received some elucidation from the inquiries of M. Dutrochet.‡ These are chiefly of importance as tending to confirm those of MM. Prevost and Dumas, who found the contraction to be produced by a doubling or folding of the fibres upon each other. M. Dutrochet regards the movement observed in some vegetables to be effected in a similar manner.

---

* *Journal de Physiologie.*

† *Dictionnaire de Medicine*, art. Generation.

‡ *Recherches sur la Structure interne des Organes des Animaux, et sur le Mecanisme de la Contraction Musculaire;* par M. Dutrochet. 1824.

MM. Prevost and Dumas, who are taking a distinguished part in the physiological inquiries of the day, have published an extended paper* on the Development of the Heart in the Fœtus, which is of interest, particularly with reference to the formation of blood, which they suppose is effected in the liver; thus agreeing in opinion with M. Edwards.

---

### PATHOLOGY.

Every thing connected with the nerves has, during the last few years, been in vogue; and we have to add to the list of writers the name of M. Martinet, who has devoted his attention to pathological inquiries, particularly to inflammation. For some interesting observations on this subject, we refer our readers to our review of M. Duges' paper (Number for December), and shall only add the conclusions deduced from M. Martinet's remarks, taken collectively: the paper itself is well worth reading. 1. That inflammation of the nerves is one of the causes of neuralgia, but among the least frequent. 2. That this inflammation has its seat, in the neurilema, &c. in the cellular tissue which unites the nervous filaments together. 3. That it has, for a constant mark, the development and increase of pain on pressing the affected nerve. 4. That this inflammation differs from neuralgia proper, which consists solely in an alteration of the sensibility of the medullary substance, in which the pain is not always aggravated by pressure, and in which it is always subject to remissions.†

In our preceding Proëmium (July 1824), we gave an account of the interesting observations of Dr. Scoutetten on the Pathological Anatomy of the Peritoneum, as far as they had at that time been carried. He has continued these, and recently brought them to a close in the "Archives Gen. de Medicine" for August.

The lesions of the peritoneum resulting from chronic inflammation, are very various, depending upon the period of the disease and the constitution of the patient. After the inflammation has lasted from fifty to sixty days, the abdominal cavity is filled with a whitish effusion, occasionally resembling milk, with numerous adventitious membranes: if these be detached, the peritoneum underneath does not appear so vascular as in acute inflammation; sometimes, indeed, it has scarcely any red colour. In some instances, the intestines are entirely glued

---

* *Observations sur le Developpement du Cœur dans le Fœtus;* par MM. Prevost and Dumas.—Bulletin des Sciences de la Soc. Phil. October and November.

† *Memoire sur l'Inflammation des Nerfs;* par L. Martinet.—Revue Medicale, Juin.

together; and very rarely is there effusion of serous fluid sufficient to distend the abdominal parietes. The omentum often becomes much thicker, and sometimes contains hydatids in its substance. In these forms of the complaint, but little pain is experienced, and pressure is borne without inconvenience; constipation is for the most part present, and a sense of weight in the abdomen. When the peritonitis has continued for a longer period, for example, for several months, disorganisations of a different kind are met with. The parietes of the abdomen are found glued to the intestines, uniting the omentum between them. This last frequently descends as far as the pelvis, where it forms adhesions, and frequently has numerous tubercles studded over it. While these are only in the earliest stages of their development, they are indicated by small whitish points on the peritoneum, more sensible to the touch than to the sight. Now and then may be perceived blood-vessels much enlarged, and radiating in a very beautiful manner. The size of the tubercles often exceeds that of a pea: they increase in number and dimensions until they coalesce; they adhere so firmly to the peritoneum, that they can with difficulty be separated from it. At first they are of very firm consistence; but, in proportion as they increase, they become softer, till their composition resembles pus, when they can easily be detached from the membrane. After having become thus soft, they again begin to harden, and occasionally attain even a calcareous induration. The interval between these tubercles assumes a variety of appearances,—its colour being red, bluish, or even black; the tubercles at the same time remaining white: along with this there is generally some, although but little, effusion.

In other instances, where the disease has lasted some months, the abdomen is distended by the quantity of the serous effusion; the intestines being pushed back, and appearing as if clustered together upon the vertebræ. The peritoneum is thickened, and presents numerous red spots, apparently produced by extravasation of blood. Occasionally large spots of ecchymosis are found; and, in this case, furrows and erosions, of various dimensions, may be perceived. The fluid effused into the peritoneal cavity differs very essentially in different cases; sometimes it appears to consist almost entirely of blood, and constitutes the *hemorrhagic peritonitis:* this generally takes place suddenly, from the ulceration of some small blood-vessels. The symptoms accompanying this form of the disease are said, by Dr. Scoutetten, to be the supervention of acute pain in a case of peritonitis which has not previously been very painful, and, along with this, acceleration of the pulse and aggravation of the general symptoms: all which phenomena suddenly disappear, the pulse becomes weak and slow, the features collapsed, and the patient feels a pleasant degree of heat in the abdomen, as if it contained warm water. Dr. Scoutetten affirms that he has met with many such cases, and that post-mortem examination has always confirmed the diagnosis.

Besides the ecchymosis above mentioned, various colours are sometimes communicated to the peritoneum, by the transudation of blood or bile. When the latter is the cause, its production is favoured by certain circumstances: thus, it takes place more readily in those who have died of acute than chronic inflammation. The blood likewise may produce a stain upon the peritoneum. Dr. Scoutetten mentions the case of a man who had thrown himself from a window, in whom nearly the whole of the serous membrane of the abdomen was red. The appearance was at first regarded as inflammatory; but more close examination led to the detection of a rupture of the spleen, from the effusion caused by which the red dye had arisen.

Gases are certainly very rarely contained in the peritoneal cavity, although some instances of this kind are recorded on good authority.* Dr. Scoutetten thinks he has once seen air contained in the peritoneum, without any organic lesion.

The fluids, independent of the effusions above alluded to, which it is said have been found in the abdomen, are bile, urine, chyle, pus, and the liquor amnii.†

A curious fact, connected with the pathology of Dyspepsia, has been ascertained by Dr. Prout,‡ and confirmed by Mr. Children,§—viz. that the acid so frequently generated during imperfect digestion, is the *muriatic.*

Rather a diffuse paper, from the pen of M. Bayle, contains some curious illustrations of anomalous Gout:‖ the nature of his observations, which are embodied in the detail of cases, prevents the possibility of quoting them, consistently with the condensed form of our present historical outline: we, therefore, in this as in other instances, give a general summary of the author's views, referring our readers to the original essay for further illustration. The results of M. Bayle's investigations are—1. That the nature of gout is unknown to us; but that it is a specific disease, probably consisting in the alteration of some of the fluids, or in the formation of a particular morbid humor. 2. Gout may affect any organ and every tissue, although it has a peculiar predilection for certain parts, which are alone often

---

* M. Portal, *Anat. Med.* tom. v.

† *Archiv. de Medecine,* August 1824.

‡ *Philosophical Transactions,* 1824.

§ *Annals of Philosophy.*

‖ *Memoire sur la Goutte anomale;* par M. A. L. I. Bayle.—Revue Med. Juin.

affected: it is, therefore, rather a general disease, than one peculiar to any organ or set of organs. 3. The symptoms by which it shows itself are inflammations, neuroses, hemorrhagies, and a variety of other phenomena, which may exist separately, successively, or alternately, according to the intensity of the malady, its regularity or irregularity, and other circumstances. 4. Numerous causes, as predisposition, delicacy of constitution, great nervous sensibility, &c. may render the diagnosis of gout extremely difficult, by giving it at one time the form of a neurosis, at another of an inflammation; and some marks are given by M. Bayle, by which we may judge between genuine instances of these and gout; but, as his diagnostic characters appear to us rather unsatisfactory, we decline giving them. 5. Notwithstanding the number of vaunted remedies for the gout, there is no real specific. None of the methods of treatment are directed against the essence of the disease—the alteration of the fluids; but each of them may be useful in fulfilling particular indications. Thus, antiphlogistics are useful where there is inflammation; antispasmodics, where it assumes a nervous form; and tonics, where there is debility, &c. Each of these means, advantageous in particular cases, would be hurtful if generally applied. "But there is one indication always presented by anomalous gout: it is, to remove it when it attacks any of the organs essential to life,—to force it, as it were, from thence, in order to bring it to the joints; and this is to be done by revulsives." Probably, the English reader will find in these remarks the reiteration of his own opinions, rather than the statement of any thing absolutely new.

It would be easy to extend this department to almost any length, did we admit any of the numerous individual cases of post-mortem examinations which have been recorded: we are very anxious, however, to be concise, and therefore confine ourselves entirely to general subjects.

---

### MEDICINE, (*including* THERAPEUTICS, *and* MATERIA MEDICA).

While Italy and France have each had their *new* medical doctrines, cultivated with enthusiasm, and supported with all the energy resulting from conviction, HAHNEMANN is the only one among the Germans who has stept from the common rank of his countrymen, to propose a system of treating diseases professedly novel, and, at all events, differing essentially from many of the received doctrines. To this system we alluded in our last Retrospect, and we now content ourselves with this brief notice, because it is our intention to return to the subject on an early occasion, and to develop his views in a more detailed manner than is consistent with the plan of the present paper.

Various papers have recently been published on the subject of Blood-letting in the cure of disease; and among these we have to notice one from the pen of the veteran HUFELAND, who enters a strong protest against the indiscriminate use of phlebotomy, likening modern physicians to some of the gods of old, who could not be appeased without blood. He reminds us that he was one of those who first advocated the antiphlogistic treatment of fever, and he now steps forward only to reprobate its abuse. Without entering into the details, we may observe that he lays down two doctrines to regulate our practice,—viz. to spare the abstraction of blood as much as possible, and to manage the crisis. With regard to this last, he seems to understand that no disease can be cured by any therapeutic means, without the interior and hidden remedial operations of nature; to effect which, the greatest quantity of blood consistent with safety is required by the system. He holds that every fever, even the most simple, may, by bleeding, be converted into one which is nervous,—particularly in epidemics. M. Hufeland further strives to prove that, in inflammations, bleeding may be carried to such an extent as to deprive the parts of the vigour necessary to effect the crisis, which he regards as necessary to recovery. However his reasoning may be received by our readers, we are satisfied there is much truth in his general objection to the use of the lancet, as now so indiscriminately employed.

A paper on the same subject, but taking a different view of it, is to be found in the Edinburgh Journal for July: it contains some judicious practical remarks.*

Some good general directions for regulating the use of blood-letting, are contained in the only other paper on this subject to which we purpose at present to refer.†

"1st. There must be either a general or local excitement, or a venous congestion, sufficient to produce either injury to the system, or considerable inconvenience and distress. This must be, at least in some measure, dependent on the tone of the arterial system, which may be either increased, natural, or even less than natural.

"2d. This must exceed the controlling power of low diet, and the ordinary febrifuges, and of frictions and the other milder means of relief; and the case must require greater promptitude than is compatible with the use of purgatives or digitalis.

---

* *Observations on the Employment of Blood-letting in the Cure of Disease.* By JAMES BONNAR.—Edinburgh Med. and Surg. Journal, July.

† *Philadelphia Journal,* May.

"3d. The importance of the relief to be procured must exceed that of the increase which venesection will produce in the debility subsequent to the termination of the disease. In this debility are included the slight dropsies which sometimes owe their cause to bleeding.

"4th. The danger, if any exist, of bleeding augmenting a subsequent debility, or a typhous disposition in the progress of the disease, or of incapacitating the patient for bearing subsequent exhausting hardships from the same cause, as in small-pox, must be of inferior consequence to the present occasion for the remedy. This is a consideration totally distinct from the last.

"5th. The excitement or congestion must be so far capable of being moderated by diminishing the force of the heart, that equal relief cannot be obtained by cups, leeches, or blisters, or not with so little injury to the system.

"In these principles there will probably be few physicians who will differ from me. They are, in fact, a mere systematic enumeration of considerations generally acknowledged and acted on.

"In the second place, when bleeding is resolved on, the quantity to be drawn must be regulated by the following principles:—

"1st. If it be desirable that a certain degree of excitement should still be kept up, and it be consequently only necessary to moderate it, faintness should, in general, be avoided; and the quantity drawn should be varied according to so many circumstances, that it would be tedious to enumerate them here.

"2d. If the object be, without the presence of the last condition, to remove or relieve prominent and dangerous, or distressing local symptoms, bleeding should be continued till some relaxation of the system be produced, as evinced by a slight degree of nausea, by muscular weakness, a swimming of the head, diminution of the warmth and redness of the skin, and, in by far the greater number of cases, by an immediate and considerable relief to the pain or other symptom to be removed.

"3d. If the symptom be of great importance,—if life itself, the comfort of the remaining portion of it, or intellect, be in danger,—and if there be no very serious grounds for fearing a dangerous or destructive prostration of the system, the patient should be bled to absolute fainting; the effect be promoted by an erect posture, if not contra-indicated; and this state preserved for a length of time, proportioned to the degree of the circumstances just enumerated.

"4th. If, however, from the prevalence of either direct or indirect debility to an alarming extent, this be forbidden, we are reduced to the ordinary rule of endeavouring to calculate the probabilities of benefit in the best manner we are able, and to act accordingly.

"5th. If it be feared, from the constitution of the patient, that fainting will occur from other causes, as it sometimes will, too soon for the removal of a sufficient quantity of blood to produce a permanent effect, and that the symptoms will recur with nearly the same violence,—the well-known causes which augment this tendency, such as heat, want of air, the erect posture, and alarm, should be removed previously to commencing the operation; and we should sometimes even set the vein bleeding a second time, after the patient has recovered from his state of depression."*

We have several times alluded to the Italian system of treating inflammation by means of large doses of tartarised antimony: we have likewise mentioned that the practice had been adopted by M. Laennec; but have not, until now, been able to specify the exact manner in which this distinguished pathologist has employed the remedy. M. Laennec generally commences with four or six grains of the tartarised antimony, dissolved in four or six half-glasses *(demi-verres)* of infusion of orange-leaves, much sweetened; he then progressively increases the dose, generally without increasing the proportion of the vehicle,—that above mentioned being chosen with a view of avoiding uausea, and guarding against the emetic properties of the medicine. Of this solution, half a glass is to be taken every two hours. The first doses generally produce evacuations, either upwards or downwards; but these soon cease if the remedy be persisted in, after which the dose may be increased to a certain point, at which (without our being able to foresee it, or to divine the cause,) the medicine will no longer be tolerated. It must then be forthwith suspended, as its use might do harm, even in very small quantities. "If," says this writer, "there were only one example of recovery from peripneumony under the use of tartarised antimony in large doses, it might with reason be attributed to chance, since no relation can be traced between the disease and the treatment,—the immediate effects of the medicine and the return to health; but, the multitude of facts obliging us to examine the matter more closely, we see with surprise ten or twelve grains of the tartarised antimony, taken for many days in succession, at first causing evacuations, and afterwards producing none. Often the disease runs its course notwithstanding, and the patient, escaping from the most imminent peril, is restored, as it were, by miracle."

* *Synoptical View of the Principles which should regulate the Employment of Blood-letting;* by B. H. Coates, M.D.—Philadelphia Journal, May.

It is of importance to know that no instance of abdominal inflammation has occurred from its use, in the practice of M. Laennec; and, when death has occurred, there has been a remarkable paleness of the mucous membrane of the stomach and bowels. It has likewise been used by him in acute hydrocephalus, in chorea, and in rheumatism affecting the joints. This practice is supported in France by the testimonies of MM. Laennec, Honoré, Double, and Ribes; and appears to us to merit the attention of our professional brethren in this country.*

The *oil of turpentine* has been set up in France as a rival to the carbonate of iron in tic douloureux; but, as yet, its reputation is supported by very unequal authority. Our readers are probably aware that M. MARTINET published a Memoir in 1823, recommending this remedy in certain rheumatic affections, particularly sciatica, and still more strongly in neuralgia.† Since this publication appeared, the medicine has been frequently tried in France, and M. DUFAUR has laid the result of his observations before the public.‡ His testimony is highly favourable, and he relates some obstinate cases in which the turpentine succeeded after other remedies had failed; in none, however, had the iron been had recourse to: and, indeed, with regard to what the French in general call sciatica, or sciatic neuralgia, as well as with regard to the cases of M. Dufaur in particular, we may observe, that they rather consist in rheumatic affections, than in the disease to which, in this country, we limit the term neuralgia. In this last, the carbonate of iron holds its high character undiminished, as may be seen by consulting the pages of this Journal through the last three or four volumes.

The *ergot*, although in disgrace among the midwives in this country, enjoys some repute in France, and considerable reputation among the Americans, whose periodicals frequently contain testimonials in its favour. Of these, the latest, and not the least respectable, is from Dr. CHURCH, who gives a short historical sketch of the drug, and informs us that he has used it, "1st, in amenorrhœa; 2d, in lingering and laborious parturition; 3d, in labours complicated with uterine hemorrhage; 4th, where the placenta has been retained, from an atonic state

---

* See *Observations sur l'Emploi de l'Emetique a haute Dose comme moyen curative, recueillies a la Clinique de l'Hopital de la Charité, et publiées avec l'autorisation de* M. le Professeur LAENNEC; par V. DELAGARDE.—Archives Gen. de Medecine, Avril.

† *Memoire sur l'Emploi de l'Huile de Terebinthe dans la Sciatique, et quelques autres Neuralgies des Membres;* par L. MARTINET, M.D. &c.

‡ *Observations de plusieurs Neuralgies, gueries par l'Emploi de l'Huile de Terebinthe;* par M. DUFAUR.—Revue Medicale, Août.

of the uterus; 5th, in retention of the placenta caused by, or accompanied with, an hour-glass contraction of the uterus; 6th, in adhesions of the placenta; 7th, as a preventive in cases where the patient has formerly suffered much from flooding, consequent to the birth of the child and extraction of the placenta; 8th, in cases of uterine hemorrhage subsequent to delivery." He gave it in doses of from ten to sixty grains of the powder; or made a decoction by gently boiling two drachms in half a pint of water for two hours, and straining; of this, one-third is exhibited every twenty minutes, till "proper" pains supervene; after which, it is discontinued. He argues that it has been condemned by Dr. RAMSBOTHAM and others in this country, without sufficient examination; and expressly states, that he "has not observed that the ergot in any case caused the death of the child.*

*Iodine* has lately been brought into notice in France, as a remedy in gonorrhœa and chronic inflammation of the testicle; complaints which M. DE SALLE calls venereal.† We should wish this term restricted to its more accurate and confined sense, since a distinction in practice must be made between lues venerea and gonorrhœa, and therefore it is as well not to confound them in theory. M. RICHON recommends the tincture of iodine to be given in doses of thirty drops twice in the day, in the latter stages of gonorrhœa; after having treated the previous inflammatory stages by leeches and the antiphlogistic regimen. In twenty-three days he effects a cure, and he has only failed in a very few instances: but it is necessary to commence with doses of twelve or fifteen drops twice in the day, gradually increasing it. We do not think that, considering the length of time admitted to be necessary for the cure by the iodine, that the practice is likely to become general.

In the treatment of buboes, after combating inflammation by the usual means, we are directed to rub the swellings with one or two drachms of the tincture of iodine several times in the day, each friction lasting about five or six minutes. The effect is a drying of the epidermis, and a sensation in the tumors sometimes amounting even to pain. Their diminution is generally perceptible in four or five days, and complete in eight or ten. M. Richon has also had some failures in these cases.

Dr. de Salle next appears, in the same paper,‡ as an advocate for the employment of an ointment, composed of the hydriodate of potash, upon the testicle, in the case of chronic en-

---

* *Practical Observations on Ergot;* by Dr. WILLIAM CHURCH.—Philadelphia Journal, May 1824.

† *Journal Complementaire,* Octobre. ‡ *Ib.* Septembre.

largement following ill-cured or long-neglected gonorrhœa; of which he details three cases. We are not quite sure, however, that these cases bear out our author in the belief that all the benefit was really derived from the iodine, since other curative means were not neglected. However, it does not appear unreasonable to suppose that iodine may produce some good where the scrofulous diathesis prevails: and it is precisely under these circumstances that gonorrhœa is frequently obstinate, and swellings of the testicles are most likely to occur.

M. Richond's Memoir upon Syphilis next claims our attention, but will not detain us long. This Memoir occupies many pages, and the opinions of M. Richond are given in the form of aphorisms, to the number of eighty-nine. To the English reader they present no novelty, and but a very confused knowledge of the subject upon which the author treats. He makes no distinction between gonorrhœa and syphilis; he mentions none of the experiments or works published in England, with the design of clearing up the natural history of the disease, though his own doctrines do no more than reiterate what has been urged in this country within the few last years, and which we have now been brought to estimate soberly and properly, with the happy result of having improved the practice in all syphilitic affections, by justly estimating the remedial virtues of mercury, and knowing when and in what degree to administer, and when to withhold it. We do not doubt that M. Richond will in a few years come to the same conclusion, if he will only follow the same guides which have hitherto directed his researches.

---

### SURGERY.

The lapse of each succeeding half-year renders it more difficult for us to collect any novelty in the art of Surgery, for what has not already been attempted or performed in the way of operation. In medical surgery, indeed, (if we may be allowed this expression,) changes are perpetually occurring, and new doctrines are continually broached; but many of these die too quickly to be noticed, and others appear so crude as to induce us to wait the result of further observation before we venture to give them additional notoriety. Yet, although we much doubt whether what we shall now have to record will tend very much to the instruction of our readers, we are quite sure that some portion of it will not fail to astonish them.

Without further preface, we proceed, then, to observe, that amputation at the hip-joint, excision of the lower jaw, of an enormous tumor, and laying open the abdominal cavity in search of a diseased ovarium, are the principal operations which we have to record. As a substitute for a dangerous operation, the breaking-down of urinary calculi, by means of Dr. CIVIALE's Lithontriptor, holds the most prominent rank. One of our continental friends also labours hard to prove that iodine is a most efficacious remedy in syphilis; another has just discovered that this disease stands in need of no remedy at all; and, to complete the climax, a young French military surgeon has, with great *sang froid*, performed the operation of lithotomy upon himself, before a looking-glass. Such is a brief sketch of the principal facts we have to relate, although we are aware that many very interesting and practical cases of surgery are recorded in the various periodical publications, but which are not sufficiently distinguished by novelty or boldness to claim a separate mention in this place.

The case of Amputation at the Hip-joint is recorded by Professor WALTHER, of Berlin, the operator in this case. The subject was a poor peasant, aged twenty-one, who had, from the age of fourteen, been suffering from deep-seated abscesses in the thigh; in addition to which, the bone was fractured in the upper part of its length, by a loaded cart passing over the limb. Suppuration in every direction followed this accident. The poor fellow seems to have had no attendance, and scarcely any proper support; and, there appearing no remedy for him but amputation, he was admitted into the surgical hospital of the university of Bonn. The following was the patient's condition when first seen by Professor Walther:

"The whole thigh was swelled and painful up to the groin, from the shaking of the broken fragments during his transport in a common cart over bad roads. A probe, passed into the fistula, ran up the thigh almost to the groin, and came in contact with the bone, which was bare. His appetite, digestion, and breathing, were good; and he was not extremely emaciated. The suppuration was profuse; the matter bloody and ill-conditioned. The ulcer in the sacrum was superficial; but, as it was impossible to put the patient in any posture by which it might be relieved from pressure, its healing could hardly be expected.

"No evident improvement was discernible at the end of six days, notwithstanding a good diet, care and cleanliness, poultices, opium, calumba, and lichen islandicus, were employed; except that the swelling of the upper part of the thigh was reduced, and the pain much lessened by the removal of some loose splinters of bone.

"The necessity of amputation was obvious, and equally so the certainty that it must be performed at the hip-joint; an operation which Professor Von Walther conceived the patient unable to support. He, therefore, prepared to dismiss him from the hospital; a resolution which the patient, gratified by the attention paid to him, heard with great regret. His mother, too, and the district physician, stated, that in that case no other fate awaited him than to lie in a stable on putrid straw, starving, and waiting, in a miserable condition, the approach of an unavoidable death; whilst, in the event of an operation, his recovery was, if not probable, at least possible. These considerations induced Professor Von Walther to change his determination, and to perform the operation on the day following."

We pause here merely to remark, that, as in only six days, the thigh was reduced, and the pain much relieved by the removal of some loose pieces of bone,—and as we are previously informed that the appetite and digestion were good, and that the patient was not extremely emaciated, we are somewhat surprised that the learned Doctor did not wait a few days longer. However, the operation was resolved upon, and was performed in the following manner:

"The patient was placed nearly horizontally, with a slight elevation of his head and shoulders, on the operating table, his breech projecting over its edge: he was kept steady in this posture by several assistants. The sound limb rested with the foot on a stool, and was also secured by an assistant: the diseased one was placed horizontally, carried outwards, and fixed by two assistants; of whom, one held the upper fragment of the broken thigh, and the other the remaining parts of the extremity.

"Another assistant, standing on the right side of the patient opposite the hip, compressed the femoral artery with the pad of a compressorium, where it passes over the horizontal portion of the os pubis. Professor Von Walther then grasped, with the whole of his left hand, a large longitudinal fold of the integuments and subjacent parts on the outer side of the hip-joint; in his right he held a large, pointed, and double-edged knife, which he thrust three inches below the anterior, superior spine of the ilium, at the outer edge of the sartorius muscle, perpendicularly through the skin, the fascia, and tensor vaginæ femoris, down to the neck of the femur. The handle of the knife was then inclined inwards, and its point passed round the neck of the femur, in a direction outwards and backwards: it thus passed in a curved line along the pit formed by the upper part of the great trochanter, and was brought through the skin three inches and a half behind that process, and about the same height as the point at which it entered. The edge of the knife was then directed in the following manner:—first, somewhat upwards, so as to clear the top of the great trochanter, and to get clearly out of the pit behind it; then perpendicularly downwards, first along the outer side of the trochanter, and then along the femur close to the bone; in the last place, at about two inches below the base of the trochanter, it was turned obliquely outwards, cutting first through the muscles, then through the integuments, and in this way completing the first flap, which was an external, and not a posterior one: it had a valve-like oval shape, and consisted of the integuments, the fascia, the greater part of the tensor vaginæ femoris, and small portions of the gluteus maximus and medius. The outer edge of the sartorius was exposed, but the muscle itself untouched.

"The first flap was raised and everted by an assistant, and at the upper part of the angle between it and the rest of the limb the capsule of the joint was seen to be exposed at its upper and outer part; and it was easily opened, when in a state of extension, by means of a large bistoury. Professor Von Walther thought it advisable to retain as much of the capsule as possible, for the purpose of covering the cavity of the joint; in the same manner as he (*Salzburg Med. Chir. Zeitung,*) and Brünninghausen (vide *Jour. of For. Med.* vol. ii. p. 70,) have proposed to retain portions of the periosteum for covering the ends of the bone in common amputations.

"In the next place, he proceeded to disarticulate the head of the bone: for this purpose the thigh, which had hitherto been kept in the line of the axis of the body, was carried horizontally inwards until it formed a right angle with it, and at the same time turned so that the toes and the front of the knee were directed upwards. To this latter point Professor Von Walther attaches much importance; for, according to him, it is only when the limb is carried into a state of adduction, so as to form a right angle with the trunk, the points of the toes being at the same time directed forwards, that the disarticulation can be effected with certainty and facility. By this method, the ligamentum teres was rendered tense, and was cut through with a small knife carried into the joint round the head of the femur; which the operator's left hand, not otherwise engaged, served to press outwards and downwards.

"A single-edged amputating knife was then carried through the joint, behind the head of the femur and round the trochanter minor, the same moment that, by a combined motion of the assistants, the limb was once more brought from a state of adduction to a nearly straight position. In order to carry the knife with facility round the head of the bone, says Professor Von Walther, it is necessary that the change in the position of the limb should be very gradually effected by the assistants, and that it should be simultaneous with the progress of the knife in the operator's hand, by whose proceeding they must be guided.

"The remaining part of the operation consisted in the formation of the internal flap. The amputation knife was carried, for the space of two inches, along the inner side of the femur: in doing so, the only caution necessary was to avoid coming in contact with the trochanter minor, with which view the edge of the knife was slightly raised as it passed along that part of the bone: in this manner all the muscles on the front, the inside, and the back part of the limb, were completely separated from the bone, whilst the femoral, nay even the ischiatic vessels, were yet undivided. An assistant then placed the thumbs of both hands in the wound, and close to the almost detached femur; whilst, with the remaining four fingers of each, he grasped the soft parts at the inner and upper part of the thigh, including the femoral artery and other great vessels; in that way commanding the flow of blood through them, and facilitating their subsequent division. Professor Von Walther advises that the assistant's hands should be applied as high as possible: first, because there the great vessels lie close together, and can be more securely compressed; and, secondly, because there is less chance of his being wounded by the knife. The amputation knife having, as already stated, been carried two inches below the trochanter minor, was directed obliquely downwards and inwards, making an incision through the muscles, vessels, nerves, and, last of all, through the skin. The formation of this internal flap, though to the patient the most painful, is to the operator (according to Professor Von Walther,) the easiest part of the operation. The latter has only to take care that it is not too small, nor yet so large as unnecessarily to increase the unavoidably great surface of the wound; that the skin is cut through sufficiently low; that the edge of the incision in it has as much a semicircular form as may be, (for perfect precision in this respect is not to be expected;) and, lastly, that the incision shall form a continuous line, without any angles or interruptions.

"The internal flap contained the largest and most important muscles of the thigh: the bulk of it was formed by the adductores, with which were connected the gracilis, pectineus, sartorius, and rectus, superiorly; and, inferiorly, the obturatores, gemelli, biceps, semitendinosus, and semimembranosus. It contained also the crural and ischiatic nerves, (of which, the latter occupied precisely the point of union of the two flaps,) the femoral artery, the profunda, the obturator, the external circumflex, the ischiatic, and numerous branches, partly of the above vessels, partly of the gluteal. After the formation of the second flap, the next step was to tie the vessels with all possible celerity, which was effected without any difficulty, the loss of blood not being greater than in an ordinary amputation. The vessels tied were the femoral artery and profunda, the femoral vein, the obturator artery, the external circumflex, the ischiatic, and six smaller ones. The femoral artery and vein were tied with a double silk thread, the other with a single one; and, according to Professor Von Walther's practice in all amputations, and in most other operations, the ligatures were cut off close to the knot.

"The external and internal flaps, in which the muscles had retracted but little, in consequence of being divided near their origin, admitted of being applied to each other without any dragging or extension, so that the surfaces mutually covered each other, and the edges of the incisions in the skin met in almost uniform contact. The internal flap, in particular, was kept up by very long pieces of adhesive plaster, of suitable width, fixed at the upper part of the hip and stretched over the buttock. The remainder of the dressing consisted of a simple superficial, retentive, and compressive apparatus, formed by coarse charpie, several compresses, and a circular bandage of appropriate breadth.

"The patient bore the operation well, and only took a few spoonfuls of wine during the time of its performance."*

The patient went on tolerably well for a few days; but the ulcer on the sacrum continued painful,—the night sweats, thirst, and fever remained,—and he died on the morning of the eleventh day after the operation. Dissection proved that all the thoracic and abdominal viscera were sound. In the amputated limb, the remarkable points are—that *the head and cervix, the trochanter major and minor, were all free from disease.* This is an important fact to keep in remembrance: it justifies the remark made by Mr. C. Bell; it might have been anticipated; and from a common amputation, if that was absolutely requisite, the patient might very probably have recovered.

Our readers will perceive that the mode of operating practised by the Professor differs both from Mr. Guthrie's and M. Lisfranc's, and we think presents some points of superiority over both. But the length of this statement prevents us from entering more deeply into this subject at present.

The records of surgery abound with histories of enormous fatty tumors, removed from various parts of the human body: in fact, such an operation is more formidable in appearance than reality; that is, if good sound surgery is employed after the excision,—if nature has fair play,—and the surface of the wound and its edges are placed in exact contact, all will do well, and that in a very short space of time.

The case that has elicited these remarks, is one related by Dr. Benedict, of Breslau: it occurred in the person of a woman,

---

* *Anderson's Quarterly Journal of the Medical Sciences*, October.

aged forty-seven; and the tumor is described as extending from the lower part of the shoulder-blade to below the sacrum, being upwards of twenty-two inches in length, and twenty in breadth. The whole tumor was moveable, and the skin (excepting at the most projecting part, where there were many varicose vessels,) was natural; there was an ulcer on the surface, produced by the injudicious meddling of a quack. There is no necessity for our describing this operation: the whole was removed, and in little more than a month the patient was entirely well, notwithstanding some sloughing took place at the upper part of the wound, and some collections of matter, requiring to be opened, at the lower part. The tumor weighed thirteen pounds.

GRAEFE and WALTHER's Journal also contains an account of the Excision of a large portion of the Lower Jaw, in the case of a female affected with an osteo sarcoma, as large as two fists. There is nothing in the operation that demands our notice particularly; and we only mention it as an additional instance of the successful result of this operation, and as introductory to a series of cases of a similar nature, published by M. DELPECH,* accompanied by reflections on the various modifications of this operation. The paper occupies forty-five pages, and is, from its very nature, incapable of much abridgment; since, in the nicety and exactness of the details, the merit of what this distinguished surgeon has advanced entirely consists. The difficulties attending the removal of the lower lip, of so great a portion of bone, and the best methods of overcoming these difficulties, are fully discussed; and M. Delpech finally informs us, that he has, upon several occasions, supplied the loss of the lip, in the Tagliacotian method, from the skin of the neck; the details of which he shortly intends to publish.

The German Journals detail the history of a very formidable operation, in which great part of the Scapula was removed, together with an enormous tumor adherent to it; the length of which was one foot, its breadth nine inches, and its projection, from the back, upwards of eight inches. The removal of this mass was undertaken by Dr. HAYMAN; and it was found that the tumor grew from the bone, so that it was necessary to remove nearly half the spine of the scapula, and the whole of the upper and inner angle: of course, great destruction of the muscles was unavoidable; and the wound was long in healing, both on account of several exfoliations, and, we suppose, from the difficulty of bringing much of the enormous cavity remaining into contact†.

---

* *Revue Medicale, Fran. et Etrang.* Oct. † Graefe and Walther's *Journal.*

In a French Journal of last July,* there are two cases of what are called Fibro-cellular Tumors detailed. The first case we only mention in order to observe upon the obstinate adherence of our neighbours to the old-fashioned and very bungling method of dressing the wounds produced by these operations. Here was a large tumor occupying the back of the neck, implicating no parts of importance, and requiring the ligature of only a few arterial branches; and yet the wound is filled with *(boulettes)* of charpie, sprinkled with colophony,—and what is the consequence? First, that the patient is seized with pain in the throat and difficulty of swallowing, obliging the surgeon to cause blood to be taken away in the night; and five weeks elapse before the wound is closed. In this case M. Dupuytren is the operator, a gentleman certainly of great attainments, a skilful and bold operator, but, we much fear, blinded by prejudice; for we cannot suppose him to be ignorant of the first principles of his art, which such a method of proceeding would at first sight seem to imply.

Of the second case, we have already given a short outline in our preceding Number: it is that of a young woman, whose death was attributed by M. Dupuytren to the sudden introduction of air into the venous system. The editors of the Journal Complementaire, who have copied this case, seem to be no less puzzled with it than our readers must be, if they take the operator's opinion as decisive: they doubt the cause of death as assigned by him, and with very great reason; they seem to insinuate that some important nerves may have been divided, or that excess of pain alone may (as has occurred, even in more simple operations,) have caused immediate death. For our own parts, we cannot pretend to explain this matter: we should have been almost tempted to think that some very important blood-vessel had been divided; but, as dissection must have discovered this, and nothing is said about it, we must leave the history as we find it, unexplained and full of difficulty as it is.

Mr. Lizars, of Edinburgh, has published a paper on Extirpation of the Ovaria, with cases.† The object of this communication is to consider the propriety of performing this operation in cases of diseased or dropsical ovaria, and the author has industriously collected together a number of instances in which it has been performed with success in France, Germany, and America. We know not whether it be to the disgrace or honour of this country, that the attempt has not, we believe, been made among us; but certainly, from the almost universally fatal

---

* *Archives Generales*, July.

† *Edinburgh Medical and Surgical Journal*, October 1824.

results that have attended the Cæsarean section in England, we do not find much encouragement to laying open the abdominal cavity; for, we presume, it will not be denied that English surgeons, if more cautious, are not less bold than their neighbours in using the knife upon proper occasions.

To return, however, to Mr. Lizars' paper, we find three successful cases occurring in the practice of Dr. MACDOWAL, of Kentucky, related at length; and then follows Mr. L.'s case, which we shall extract.

"In the year 1821, I was requested by my friend, Dr. Campbell, lecturer on midwifery, to visit a woman with an abdomen as large as if in the ninth month of gestation. On examination, the tumor occupied the whole abdominal cavity, and appeared to roll from side to side; the uterus per vaginam felt natural, and her catamenia had been regular, but caused excruciating pain when they occurred. She stated that she was twenty-seven years of age, had born only one child, and in twelve months afterwards had a miscarriage; two or three months after which, towards the end of 1815, she became sensible of a considerable enlargement of her belly, that began on the left side, and which she attributed to several blows and kicks received from a brutal husband, from whom she was now separated; that her neighbours now abused her, and made such complaints to her employers, that they dismissed her. At that time she earned, and now earns, her livelihood by binding shoes. Being now without the means of support, she applied to a county hospital, but was in a few days dismissed, on the supposition of being with child. She then consulted a number of respectable practitioners, but all of them cruelly taunted her with being pregnant. At the end of two years, she perceived a small moveable swelling in her left groin, which she allowed to increase for twelve months, when she came to Edinburgh, and, on consulting a surgeon, he opened it with a lancet, and discharged a large quantity of thin matter. On examination, this was a lumbar abscess, which she ascribed to a fall on her back three years previously. The evacuation of this fluid did not in the least diminish the magnitude of the abdomen; and she imagined she could distinguish between the pain of the lumbar abscess and that of the tumor in the abdomen. She was admitted into the hospital of this place, and remained for thirteen weeks, without receiving any relief. She consulted the chief medical gentlemen of this city, many of whom pronounced her pregnant, and all of them tried to dissuade her from an operation. Two put her under different courses of mercury; and, after a consultation, one punctured the abdomen for dropsy of the ovarium.

"Before having recourse to the operation of gastrotomy, I deemed it my duty to have the opinion of the principal practitioners of this city, either by personal consultation, or by sending the patient to them. The woman herself also had previously waited on many of them. Some said, that to operate would be rash; others, that I would kill my patient. It was agreed by all, that there was a disease of one or both ovaries; and she had been twice tapped for dropsy of the left ovary, the result of a formal consultation of some of the ablest medical men of this city. Convinced, from the history of the disease in the records of medicine, and from gastrotomy having been successfully performed for volvulus, and from the Cæsarian section, that there was little to apprehend either from loss of blood or peritoneal inflammation, I felt desirous to endeavour to relieve the woman by an operation; but was anxious to have the sanction of some other surgeon or physician besides my friend, Dr. Campbell, who at once offered to assist me. All whom I took to see the patient, and all to whom I sent her, said that the disease was an affection of the ovarium; but all of them condemned an operation. My patient, therefore, abandoned to her gloomy condition, called on me repeatedly, urging me to try the operation, otherwise she would do it herself. At last, as her pain became perfectly intolerable, and she was still urgent, I resolved to operate. During the preceding period, I had directed my attention to the lumbar abscess, and applied caustic eschar after eschar.

"Wednesday, 24th October, 1823, was the day appointed for the operation; therefore, on the day preceding, she took a dose of the compound powder of jalap, which operated also on Wednesday morning, so as to preclude the necessity of administering an enema; she also made water immediately before, in order to empty the bladder. The emptying of the rectum by a glyster, and the drawing off the urine, or taking care that the patient makes water, are circumstances of some consequence to be attended to, in all operations of the abdominal cavity. As inflammation appears to be induced generally by exposure to cold, and as these cases succeeded so well in America, I desired the room to be heated to 80° Fahrenheit. When the temperature of the room had arrived at this heat, I placed the patient on a table covered with a mattress, and two pillars supporting her head, and commenced the operation, in the presence of Dr. Campbell, Dr. Vallange, late surgeon of the 33d regiment, Mr. Bourchier, surgeon of the 36th regiment, and several other medical gentlemen, by making a longitudinal incision, parallel with and on the left side of the linea alba, about two inches from the ensiform cartilage, to the crista of the os pubis, through the skin and cellular substance, when the peritoneum appeared, the recti muscles being separated by the distension consequent on the present disease and former pregnancy. I then made a small incision through the peritoneum, introduced a straight probe-pointed bistoury, and made a more extensive opening, into which I inserted the fore and middle fingers of the left hand, so as to direct the instrument, and to protect the viscera. With this instrument I made the internal to correspond with the external incision; while my friend, Dr. Campbell, who assisted me, endeavoured, but in vain, to confine the intestines within the abdominal parietes. Apprehensive of peritoneal inflammation, of which many said my patient would die, I enveloped the intestines in a towel dipped in water about 98°. I now proceeded to examine the state of the tumor, when, to my astonishment, I could find none. I next requested Drs. Campbell, Vallange, and Bourchier, to make themselves satisfied that there was no tumor; when Dr. Vallange observed, that he felt a tumefaction on the left side of the pelvis. This, on investigation, was found to be a flattened tumor of no great magnitude, at the left sacro-iliac synchondrosis of the pelvis, lying beneath the division of the common iliac artery into its external and internal branches. Having satisfied all present that this was not the tumor which was anticipated,—that it was impracticable to extirpate it,—and that the uterus and ovaria were perfectly sound and healthy, I proceeded to return the intestines, and to stitch up the wound, carrying the needle as deep as possible, and applying straps of adhesive plaster between the stitches. Compresses of lint were next laid along, and the nine-tailed bandage bound round the body. I then carried her to bed, and gave her an anodyne draught of forty drops of laudanum, which was almost immediately rejected. Ordered her warm toast-water and tea.

"When the intestines protruded, and baffled all the efforts of Dr. Campbell and the other gentlemen to confine them, I shall never forget the countenances of my pupils and the younger members of the profession. This fact of the intestines being forced out, proves, along with others, that the lungs can be expanded, although atmospheric air be admitted into the abdominal cavity: the diaphragm acted with great vigour, and with powerful impetuosity. The operation was performed at one o'clock of the day, and by seven in the evening she had vomited twice; had flying pains in the abdomen, a little hurried breathing, pulse at 100, and some thirst; she also felt uneasiness from inability to void her urine, which was drawn off by the catheter; and, as a precaution, I bled her to syncope, which occurred when eleven ounces were abstracted. She lost little or no blood during the operation."

This woman perfectly recovered. Vomiting occurred for two or three days after the operation, attended with much pain in the abdomen and fever; but, by prompt and large bleedings, and a strictly antiphlogistic plan, these symptoms were subdued, and she now gains her livelihood as usual.

The next case is one of Ovarian Dropsy, in a woman thirty-six years of age, who had been repeatedly tapped. After one of these operations, the canula was suffered to remain in the wound, and a plug was inserted into its mouth, and the fluid was drawn off several successive times, with an interval of eight days between each; when no good being obtained, diluted port wine was injected in one instance, and a solution of sulphate of zinc in the other; both of which merely produced a sensation of heat whilst they remained in the cavity. The woman, however, sunk under the disease, but not for many weeks after these operations.

In our last Retrospect, we noticed a mode of performing amputation by Messrs. LISTON and SYME, which did not differ materially from the operation with two flaps, as described by DESSAULT. We have, in the same Journal for the month of July, some observations by Dr. CREASER upon the same subject. This gentleman, who was for many years surgeon to the Bath Infirmary, recommends the operation by a double flap; but his mode of performing it differs from that described by Messrs. Liston and Syme: the difference consists in his making the incision from without inwards, dissecting the skin for about half an inch, and then dividing the muscles at the retracted edge. He says, that this mode of performing the operation saves pain to the patient,—but this to us appears doubtful; for, granting that the pain is somewhat greater in the first instance, by thrusting a pointed knife through the limb, yet it must be recollected that, in Dr. Creaser's method, there are in fact four incisions: the skin is first divided anteriorly, then the muscles, and the same is repeated at the posterior part of the limb. We doubt not that, in either way, the stump is much better than in the common mode of performing this operation; and, in the thigh especially, it is an object of paramount importance to have the bone well covered. Dr. Creaser also gives a sketch of the knife which he has been in the habit of using; its blade is only five inches in length, its breadth half an inch, and towards the point there is a convexity, as in a common scalpel.

We might, perhaps, pass over entirely the operation of Lithotomy as performed by M. CLEVER upon himself;* but that, as a point of curiosity, it demands notice, and on one account may be instructive,—though we hope and believe not to any

* *Journal Universel*, Octobre.

English surgeon. The story of this case is as follows:—M. Clever had suffered from the stone from his infancy, and had undergone the operation five times, and was now enduring, for the sixth time, the torments of this painful malady: he, therefore, determined upon immediately relieving himself; and placing himself before a looking-glass, and raising the scrotum with one hand, he plunged the point of a bistoury perpendicularly into that part where the operation is usually performed; after resting a moment, he prolonged the incision through the integuments, and put his finger into the wound, hoping to feel the stone, but he found the division he had made imperfect; he therefore again had recourse to his bistoury, and completed the section: then, with the assistance at first of one finger, and afterwards of two, he searched for and extracted a stone as big as a large nut. The urine flowed in abundance; he dressed his wound, and slept soundly. The next day, M. Clever informs us, he was as gay as if nothing had happened. Many medical men, and others whom he knew, astonished at the account they had heard, went to see him, in order to be assured of the fact; and among the number was Professor Beclard, who examined the stone. M. Clever recovered perfectly. The stone, when examined, appeared to owe its formation to a piece of sponge-tent, with which the wound in the former operation had been plugged. And this is the practical lesson to be deduced from the story: for what purpose could be answered by putting sponge-tent into a wound after lithotomy? Did the surgeon fear the healing of the external wound before the bladder had united? But we need not make any comment upon such practice.

Whilst speaking upon lithotomy, it will, of course, be expected that we should say something of Dr. CIVIALE's instrument for breaking down calculi in the bladder. The instrument has been introduced into this country, and a trial has, we understand, been made with it; but the operator, we are told, did not succeed in grasping the stone, although the instrument was introduced without much difficulty, and the quantity of pain produced was not very great. In Paris, we have been given to understand that Dr. Civiale's success has not been of late so great as was at first promised; that great irritation and pain has been produced by the action of the instrument; and that death even has appeared to have been hastened by its use. We trust that we shall not be accused of endeavouring to raise any overstrained objections to the employment of this instrument, of which we have had no experience; but it does appear to us that the operation of a mechanical contrivance to seize, drill, and break a calculus in the human bladder,—an operation which must be frequently repeated, perhaps for half a year, before the object is achieved,—must give rise to a degree of irritation likely to incur the risk of life; especially as the subjects in which it is recommended to be employed are those in whom the common operation for lithotomy, either on account of diseased prostate or bladder, is rendered more than usually hazardous. Besides, should the whole of the calculus not be reduced in such a manner as to enable every particle to be expelled from the bladder, what has the patient gained, but a very temporary respite?

The operation for lithotomy performed by the lateral method, is in general tolerably successful in private practice; at least, it appears to us to be looked upon in too formidable a light. Mischief, instead of good, has occurred from so many changes of instruments and methods of proceeding, as have lately from time to time been proposed; and we would wish to ask, what are the real objections to an operation, which has been so very generally successful, in the hands of the late Mr. Cheselden, of the present Mr. Martineau, of Mr. Dalrymple, and many other surgeons, both of London and the country; and how, under any improved plan, could greater success be attainable?

### PHARMACY.

The only article which our limits will allow us to give in this department, is a very useful table, recently published by M. Fontanelle, and which we take from the pages of a respected contemporary;* with this change, however, that we have altered the terminations of the alkaline vegetable principles from *ine* to *a*, as being more suited to the English language.

### SYNOPTICAL TABLE

*Of the Physical, Chemical, Medicinal, and Deleterious Properties of the immediate Vegetable Principles, and of the Alcoloids recently discovered.* By M. Julia Fontanelle, &c.

| NAME of the Substances, of their Discoverers, and their natural Conditions. | Physical Properties. | Chemical Properties. | Medicinal and Deleterious Properties. |
|---|---|---|---|
| 1. Atropia.—Discovered by *Brandes* in the atropa belladonna. | Inodorous, insipid; crystallises in prismatic needles; colourless; turns green the syrup of violets. | Is decomposed by the action of caloric; almost insoluble in water, alcohol, and ether; neutralises much acid and produces salts, the watery solutions of which give out, by evaporation, vapours which dilate the pupil and act as narcotics. | Is the deleterious principle of the belladonna. |

* *Medical Repository*, November.

| NAME of the Substances, of their Discoverers, and their natural Conditions. | Physical Properties. | Chemical Properties. | Medicinal and Deleterious Propeities. |
|---|---|---|---|
| 2. BRUCIA.—Discovered, in 1819, by *MM. Pelletier* and *Caventou*, in the false angustura, in the state of a gallate. | In leaf-shaped masses, of a pearly white, having the appearance of the boric acid; and sometimes in allongated prisms; inodorous, bitter, acerb, and acrid; turning the syrup of violets to green. | Fusible at 102° Centigrade, congealing as it cools; is decomposed by a higher temperature; inalterable by the air; soluble in 500 parts of boiling water, and in 850 of cold; remaining in the state of a hydrate, and retaining one-fifth of water; soluble in alcohol, almost in all proportions, and but little so in ether; forms salts, which are poisonous, bitter, crystallisable, and which may be decomposed by morphia. Is turned to a crimson red by concentrated nitric acid. This colour is changed by caloric to yellow: in this state the hydrochlorate of tin forms with it a beautiful violet precipitate, a character which is peculiar to brucia. It is composed of 75.04 of carbon, 11.21 of oxygen, 7.22 azote, 6.63 of hydrogen. | This poison acts on the spinal chord, producing tetanic contractions of the muscles. May be given in paralyis, from a dose of one-eighth of a grain to five grains in the day. Four grains is sufficient to kill a rabbit. |
| 3. CATHARTIA.— Discovered, in 1820, by *MM. Lassaigne* and *Feneulle*, in the follicles and leaves of senna. | Solid; brownish yellow; odour peculiar; bitter and nauseous. | Attracts humidity from the air; is decomposed by caloric; is very soluble in water, alcohol, and ether; is decomposed by the concentrated acids. | Is the cathartic principle of senna. |
| 4. CINCHONIA.—Disc. by *Duncan*, in cinchona bark. | White; crystallisable in needles; inodorous, bitter; turns the syrup of violets to green. | It is partly decomposed and partly volatilised by caloric; it burns without leaving a residuum. In contact with the air it passes, in time, into the state of a carbonate; soluble in 25.00 parts of boiling water; soluble in alcohol, very little so in ether; saturates more acid than quina, and forms, with acetic acid, an uncrystallisable salt, yet the acetate of quina crystallises. It is composed of carbon 76.97, oxygen 7.97, azote 9.02, hydrogen 6.22. | Its medicinal properties are the same as those of quina, with this difference, that it takes five times as many parts, by weight, of cinchonia to produce the same effects as follow one part of quina. It is almost never used. |
| 5. CYSTISIN.—Disc. in 1820, by *MM. Las-* | Solid; yellowish green; taste disagreeable. | Decomposed by heat, as also by concentrated acids; very soluble in water, alco- | In the dose of eight grains it occasions vertigo, cramp |

| NAME of the Substances, of their Discoverers, and their natural Conditions. | Physical Properties. | Chemical Properties. | Medicinal and Deleterious Properties. |
|---|---|---|---|
| *saigne* and *Chevallier*, in the seeds of the *cystissus laburnum.* | | hol, and ether. It contains no azote. | of the stomach, and vomiting, which sometimes continue for two hours. |
| 6. DELPHIA. —Disc. in 1819, by *MM. Lassaigne* and *Feneulle*, in the seeds of stavesacre (*delphinium staphysagria.*) | A whitish, crystalline, and moist powder; inodorous; acrid and very bitter; turns syrup of violets to green. | Fusible, and becomes, as it cools, a hard and brittle substance; at a higher temperature it is decomposed, and burns without residue. Nearly insoluble in water, but very soluble in alcohol and ether. It forms neutral salts, which are acrid, very bitter, and very soluble. Concentrated nitric acid gives a yellow tint to this alcali, whilst the same acid gives a reddish colour to brucia and morphia. Not analysed. | Its effects are much more energetic than those produced by the stavesacre. Six grains kill a dog in the space of two or three hours. In the state of an acetate, it is still more poisonous. |
| 7. DIGITALIA. —Its existence declared by *MM. Lassaigne* and *Chevallier*; and actually discovered by *M. Le Royer*, in 1824, in the leaves of *digitalis purpurea.* | Microscopic crystals, in straight prisms, with rhomboidal bases; inodorous; very bitter; turns syrup of violets green. | Decomposed by caloric; very deliquescent; very soluble in water, alcohol, and ether. The action of acids on it has not yet been well ascertained. | Half a grain dissolved in two drams of lukewarm water, and injected into the veins of a cat, killed it in a quarter of an hour, without occasioning any appearance of pain. One grain and a half, injected into the jugular vein of a dog, produced the same effect. The blood of animals thus poisoned assumed a venous tint, and a slight tendency to coagulate. |
| 8. EMETIA.— Disc. in 1817, by *M M. Pelletier* and *Magendie*, in the roots of the *viola emetica, callicocea ipecacuanha,* and *psycotria emetica;* and by *M. Boullay* in the violet. | When pure, it is in a white powder; inodorous, and slightly bitter; turns syrup of violets green. | Fusible at 50° Centigrade; above that it is decomposed. It becomes opaque on exposure to the air; little soluble in water, but very soluble in alcohol and ether. It produces, with acids, crystallisable salts, with excess of acid only, which are decomposed by the alcalies and magnesia. Galls precipitate it in flocculi of a dirty white. It is composed of carbon 64.57, oxygen 22.95, azote 4.00, hydrogen 7.77. | It is the emetic principle of ipecacuanha. Its activity is such, that one-16th of a grain produces vomiting. The dose is one grain for adults. In that of two, it has killed a dog. |

| NAME of the Substances, of their Discoverers, and their natural Conditions. | Physical Properties. | Chemical Properties. | Medicinal and Deleterious Properties. |
|---|---|---|---|
| 9. GENTIANIN. — Found by *MM. Henry* and *Caventou*, in the root of gentian. | In small yellow needles; inodorous, and very bitter. | By the action of caloric, part of it sublimes in yellow needles, and the rest is decomposed; is not changed by the action of the air; soluble in water, communicating to it a strong bitterness; very soluble in alcohol and ether; soluble in acids. (The sulphuric acid carbonises it.) | A very energetic tonic and febrifuge. |
| 10. IODINE.— Disc. in 1813, by *Courtois*, in sea-water, in some fuci, and in several kinds of impure soda. | In species of bluish laminæ; odour, that of cloruret of sulphur. A simple body. | Fusible at 107° Centigrade; it boils at 175°, and escapes in violet-coloured vapours; is not changed by the air. Soluble in 7000 parts of water; soluble in ether, and the more so in alcohol the more it is rectified. These solutions change the colour of the skin to yellow. With oxygen it forms the iodic acid, and with hydrogen the hydriodic acid. | Used in the treatment of goître, of scrofula, of chlorosis, of cancer of the womb, of white swellings, tumors, scrofulous phthisis, inveterate syphilitic indurations of the glands, &c. It is employed in substance, in an alcoholic tincture, in the state of a salt, and as a pomade. In the dose of from one drachm to one and a half, it proves fatal, and produces ulceration of the mucous coat of the stomach. |
| 11. LUPULIN. —By *M. Planche* and *MM. Ives, Payen*, and *Chevallier*, in the yellow matter of the flowers of hops. | Solid, of a yellowish white; aromatic, and very bitter. | Decomposed by caloric; is not changed by air; soluble in water, alcohol, and ether. | Tonic and narcotic, not poisonous. Doses not yet determined. |
| 12. MORPHIA. —Disc. in 1817, by *Serturner*, in opium, in the state of a meconiate. | White crystals in triangular prisms; inodorous; insipid; solutions bitter. Turns syrup of violets green. | Melts and is decomposed at a slightly elevated temperature; unchanged by the air; insoluble in cold water; soluble in 42 parts of cold alcohol, and in eight of ether. Its salts are bitter. Nitric acid turns it red; hence it is an important test of the presence of this substance. Morphia is composed of carbon 71.01, oxygen 17.26, azote 5.177, hydrogen 6.533. | Narcotic, without the inconveniences of opium. M. Bally thinks it is as active as its acetate, to which the preference is given, on account of its solubility. As this salt is difficult to crystallise and to preserve, the sulphate appears preferable. The dose of these two salts is from one-fourth or one-third of |

| NAME of the Substances, of their Discoverers, and their natural Conditions. | Physical Properties. | Chemical Properties. | Medicinal and Deleterious Properties. |
|---|---|---|---|
| | | | a grain a-day, in pills or in a syrup, to four grains a-day. It is a poison which has lately attracted much notice. |
| 13. NARCOTIA.—In 1802, constituting 1-50th of opium, by *M. Derosne*, who named it *opiane*. | In delicate needles, or in rhomboidal prisms. Inodorous and insipid. | It melts like fat, and is decomposed; unchanged by the air; slightly soluble in water; soluble in 24 of boiling alcohol, or in 100 cold; soluble in cold ether; forms poisonous salts. Composed of carbon 68.88, oxygen 18.00, azote 7.21, hydrogen 5.91. | Seems to act as a narcotic. M. Bally has given it to the extent of 60 grains, without obtaining very remarkable effects from it. Its acetate, in the dose of a grain, has killed a dog. |
| 14. PICROTOXIA. — Disc. by *Boullay* in the *coccus orientalis* (the fruit of the *menispermum cocculus.*) | In quadrilateral white prisms. Inodorous, very bitter. Turns syrup of violets green. | Decomposed by caloric; unchanged by the air; soluble in 25 parts of boiling water and 50 of cold; soluble in three of alcohol, and very soluble in ether; forms very bitter salts, which are but little soluble in water. | It is to this substance that the coccus orientalis owes its deleterious properties. Three or four grains of it kills the largest dog in an hour. It is also a most active poison on fish. |
| 15. PIPERIN. — Discovered by *Œrstaed* in black pepper. | In colourless and translucid prisms. Inodorous, and almost insipid. | It melts and is decomposed by heat; unchanged by the air; insoluble in cold water, very slightly soluble in boiling water; very soluble in alcohol and ether. | According to Bertini, Meli, Micclini, Simoneti, and Simiscalchi, it is a most energetic febrifuge, and in a much smaller dose than the sulphate of quina. It may be carried as far as 24 grains in the 24 hours. |
| 16. QUINA.—In 1820, by *MM. Pelletier* and *Caventou*, in the cinchona. | White and incrystallisible; inodorous; very bitter. Turns syrup of violets green. | It melts, becomes idiolectric, and assumes, by friction with woollen cloth, the resinous electricity; is unchangeable in the air; soluble in 700 parts of water; soluble in alcohol, and still more so in ether; forms very bitter salts, of a pearly hue. Saturates less acid than the cinchonia, and gives a crystallisable acetate. Is composed of carbon 74.56, oxygen 7.7, azote 10.84, hydrogen 6.90. | Its virtues the same as the cinchona bark, but much more energetic. The sulphate of quina is preferred. Four grains of this salt is equal to two drachms of the bark. This alcali and its salts are five times more energetic than the cinchonia and its sulphate. |

| NAME of the Substances, of their Discoverers, and their natural Conditions. | Physical Properties. | Chemical Properties. | Medicinal and Deleterious Properties. |
|---|---|---|---|
| 17. **Rhubarbarin.** — Disc. by *Pfaff* in the rhubarb cultivated in Europe. | Solid; dark brown; opaque; odour disagreeable; taste bitter and nauseous. | Decomposed by heat; deliquescent; very soluble in water, alcohol, and ether. Nitric acid converts it into oxalic acid. | It appears to be the medicinal principle of rhubarb. |
| 18. **Scillitin.** —By *Vogel*, in the bulbs of the *scilla maritima.* | White, brittle, transparent; fracture resinous; inodorous, and taste bitter. | Decomposed by heat; deliquescent; very soluble in water, alcohol, and ether. The action of acids not yet ascertained. | The medicinal principle of the squill; it excites vomiting, the secretion of urine, and diarrhœa. |
| 19. **Solania.** —In 1821, by *Desfosse*, in the *solanum dulcamara* and in the *boletus esculentus.* | White, grained, or in quadrilateral microscopic prisms: inodorous; slightly bitter and nauseous. Turns syrup of violets green. | Decomposed by heat; unchanged by the air; insoluble in cold water; soluble in 8000 parts of boiling water; little soluble in alcohol and ether; forms incrystallisable and bitter salts, which, by evaporation, form a gummy mass. | Excites vomiting and sleep. Its acetate has only been as yet tried. Its effects have been little attended to. |
| 20. **Strychnia.**—In 1818, by *MM. Pelletier* and *Caventou*, in the *nux vomica*, and the bean of St. Ignatius. | In a white, opaque, and sometimes pearly powder; inodorous; bitterness insupportable. Turns green the syrup of violets. | Neither fusible nor volatile; unchanged by the air; soluble in 6667 parts of water at 10° R., or 2500 of boiling water. Its solutions have an orange colour; a solution of one 600,000th part of a grain imparts a bitter taste; very soluble in alcohol; insoluble in ether. Forms some crystallisable salts, more poisonous than the strychnia. Composed of carbon 78.22, oxygen 6.38, azote 8.92, hydrogen 6.54. | This is the most active poison of all the vegetable alcaloids. It is twelve times more active than brucia; one-8th of a grain kills the strongest dog, and one-4th of a grain produces on a healthy man very sensible effects. |
| 21. **Veratria.** —In 1819, by *MM. Pelletier* and *Caventou*, in the cevadilla, white hellebore, and in the colchicum. | A white, inodorous powder; taste acrid and burning. Turns green the syrup of violets. | Fusible at 50° Cent.; on cooling it presents a translucid mass of an amber colour; unchanged in the air; soluble in 1000 parts of boiling water; very soluble in alcohol; insoluble in ether. Its salts are incrystallisable. Composed of carbon 66.75, oxygen 19.60, azote 5.04, hydrogen 8.54. | A dangerous sternutatory. In a very small dose, it produces vomiting and copious alvine evacuations. |

